# Suppressed catalytic efficiency of plasmin in the presence of long-chain fatty acids

**DOI:** 10.1111/j.1742-4658.2008.06288.x

**Published:** 2008-03

**Authors:** Anna Tanka-Salamon, Kiril Tenekedjiev, Raymund Machovich, Krasimir Kolev

**Affiliations:** 1 Department of Medical Biochemistry, Semmelweis UniversityBudapest, Hungary; 2 Department of Economics and Management,Technical UniversityVarna, Bulgaria

**Keywords:** arachidonate, Monte Carlo simulation, oleate, progress curves, stearate

## Abstract

Thrombi, which are dissolved primarily by plasmin (EC 3.4.21.7.), contain up to millimolar concentrations of fatty acids and these are known to affect the action of the protease. In the present study the modulation of plasmin activity was characterized quantitatively in a continuous amidolytic assay based on synthetic plasmin substrate (Spectrozyme-PL). A novel numerical procedure was applied for identification of kinetic parameters and their confidence intervals, with Monte Carlo simulation of the reaction progress curves, providing adequate grounds for discrimination of different models of the enzyme action. All three fatty acids caused a 10–20-fold increase in the Michaelis constant on Spectrozyme-PL (baseline value 5.9 μm). The catalytic constant decreased from 5.8·s^−1^ to 2.4–2.8·s^−1^ in the presence of arachidonate and oleate, but increased to 14.8·s^−1^ in the presence of stearate, implying enhancement of plasmin activity at saturating substrate concentrations. However, based on the ratio of the catalytic and Michaelis constants, all three fatty acids acted as inhibitors of plasmin with various degrees of potency, showing concentration dependence in the range of 10–65 μm for oleate and arachidonate, and 115–230 μm for stearate. The reported effects of the three fatty acids require the presence of kringle 5 in the structure of the protease; miniplasmin (des-kringle 1-4 plasmin) is as sensitive to fatty acids as plasmin, whereas the activity of microplasmin (des-kringle 1-5 plasmin) is not affected.

The dissolution of intravascular thrombi is performed through the hydrolytic degradation of their fibrin matrix, a process catalyzed by the serine protease plasmin (EC 3.4.21.7.) [1]. Arterial thrombi contain millimolar concentrations of phospholipids [2] and free fatty acids [3], which presumably originate from the highly compacted platelet content of the thrombi [4]. These lipid constituents of thrombi profoundly modulate the fibrinolytic process [2,3,5–7]. In the few studies evaluating the effect of long-chain fatty acids on plasmin activity, both stimulation [5,7] and inhibition [3,6,7] have been reported, but the exact kinetic characteristics of plasmin in the presence of different fatty acids are still unexplored. It was therefore of interest to examine the effects of various potentially relevant fatty acids on plasmin. The three most abundant fatty acids in the structure of platelet membranes are arachidonic acid, stearic acid and oleic acid, representing 22.0, 19.5 and 18.8%, respectively, of the total fatty acid content of platelet phosphoglycerolipids [8]. Accordingly, the present study was undertaken with these three fatty acids, expanding on our recent report [3] in which oleic acid was used to model the modulatory effects on the fibrinolytic system.

## Results and Discussion

### Influence of fatty acids on the plasmin amidolytic assay

**Fig. 1:**
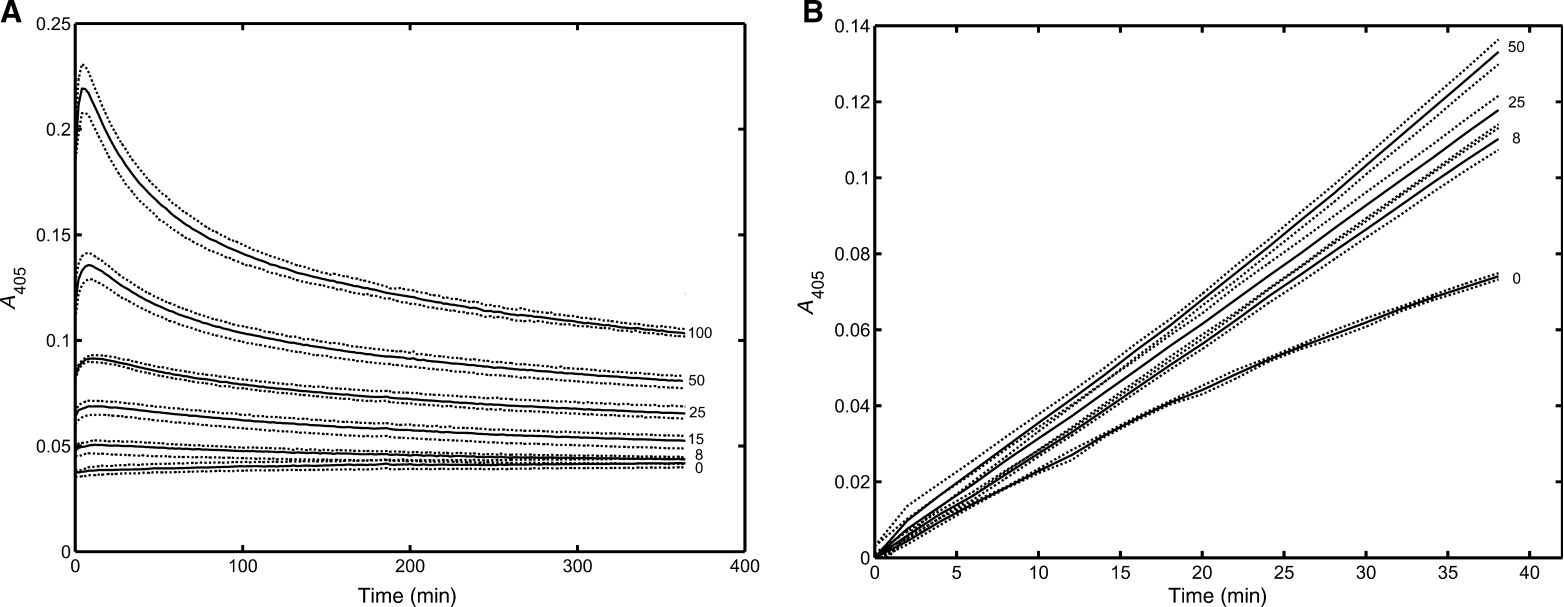
Light attenuation of oleic acid micelles in the presence of synthetic plasmin substrate. (A) Twenty microlitres of 1 mm Spectrozyme-PL was added to 180 μL of various concentrations of oleic acid (the numbers next to the lines indicate the concentration in μm) and the absorbance at 405 nm was measured. (B) Twenty microlitres of 1 mm Spectrozyme-PL was added to 180 μL of 1 nm plasmin solution containing oleic acid, at concentrations (in μm) indicated by the numbers, and the absorbance at 405 nm was measured. The mean and standard deviation (dotted lines) of five measurements are shown for both panels.

For determining the exact kinetic parameters of plasmin we chose a simple amidolytic assay based on a synthetic plasmin substrate (Spectrozyme-PL). When the plasmin amidolytic activity assay was performed in the presence of free oleic acid, some spurious effects were observed (Fig. 1). The optical phenomenon illustrated in Fig. 1A was not wavelength specific (because the same absorbance changes were seen at 340 nm; data not shown) and was not related to substrate breakdown (because plasmin released all the expected amount of *p*-nitroaniline after the incubation, as shown in the figure). Thus, these optical changes can be attributed to the formation of fatty acid micelles and their re-arrangement effected by Spectrozyme-PL. The results presented in Fig. 1B were observed if relatively weak plasmin activity (e.g. 1 nm plasmin) was superimposed on the initial phase of the optical changes in Fig. 1A and these can be misinterpreted as an activating effect of oleic acid. Therefore, in the present study we chose to work with higher plasmin concentrations (20 nm) and water-soluble sodium salts of the fatty acids, the turbidity effects of which are smaller compared with the free acid and are not influenced by the plasmin substrate.

### Analytical models of plasmin inhibition

**Fig. 2:**
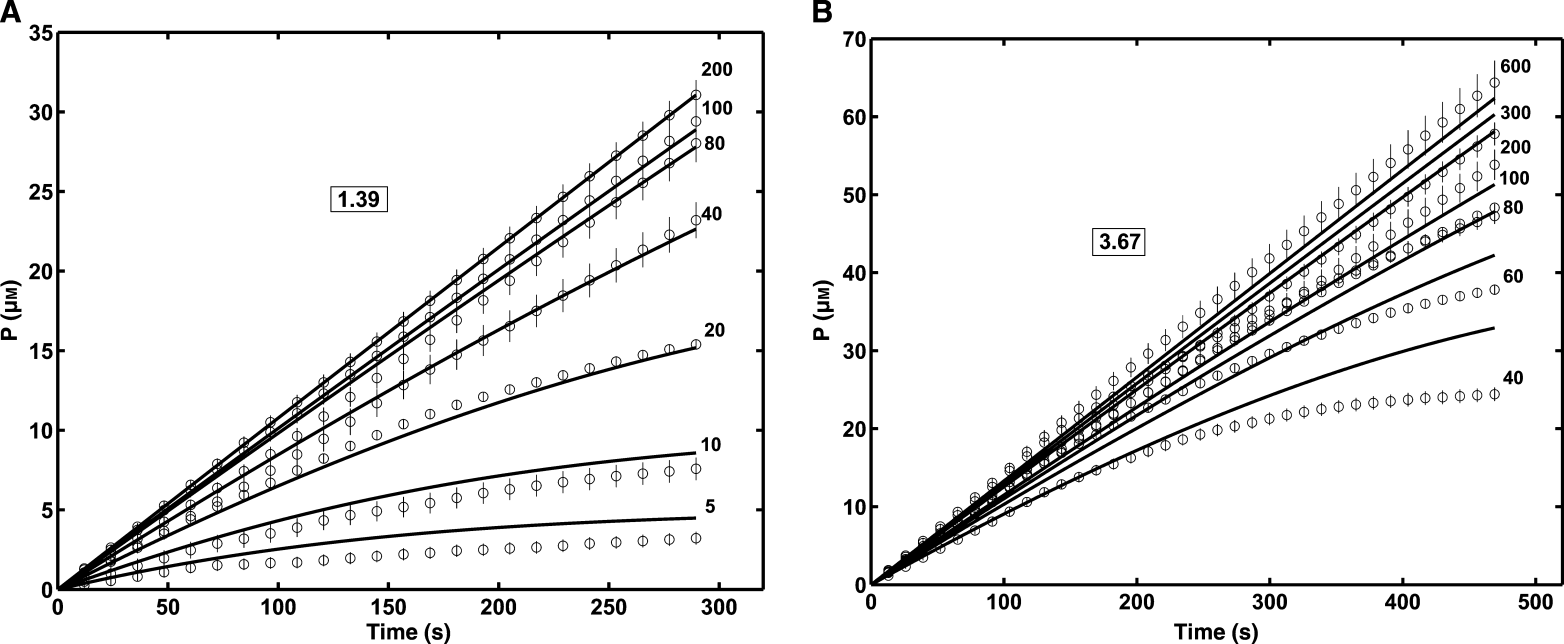
Amidolytic activity of plasmin in the presence of oleate and stearate. The hydrolysis of Spectrozyme-PL (the concentrations, in μm, are indicated at the end of the curves) by 20 nm plasmin was monitored in reaction mixtures containing 10 μm oleate (A) or 115 μm stearate (B). The *p*-nitroaniline (P) generated is presented as the mean (symbols) and standard deviation (cross-lines) of four measurements. Lines represent the best global fit of the data set to the equation of Model I described in the Materials and methods. The measure for goodness-of-fit (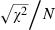 where *N* is the total number of measurement points) is presented by the numbers in boxes.

**Fig. 3:**
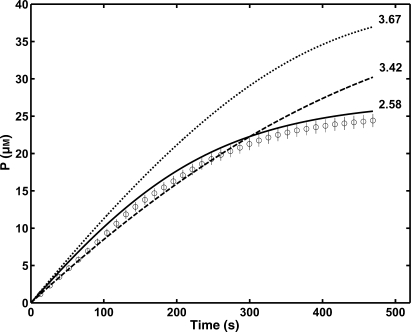
Comparison of three models for the catalytic action of plasmin in the presence of stearate. The *p*-nitroaniline (P) released in the course of hydrolysis of 40 μm Spectrozyme-PL by 20 nm plasmin in the presence of 115 μm stearate is shown by symbols (mean ± standard deviation of four measurements). Lines represent the curve for the 40 μm substrate concentration from the best global fit of the data in Fig. 2B to the equation of Model I (dotted line), Model II (dashed line) and Model III (solid line), as described in the Materials and methods. The numbers next to the curves indicate the goodness-of-fit (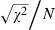) for the parameter optimization procedure, according to the respective model.

**Fig. 4:**
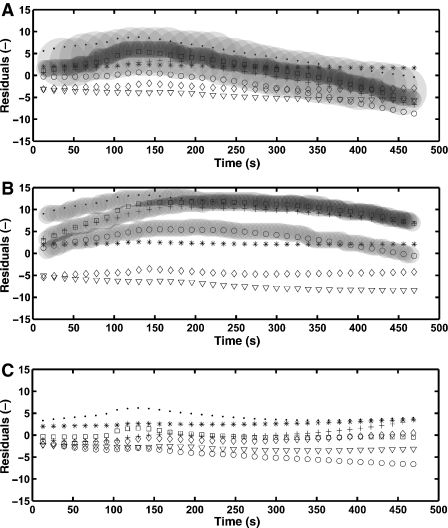
Residual plots for the discrimination of three models of plasmin action in the presence of stearate. Residual values 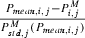
were calculated using the measured *P*_mean,i,j_ values and their model standard deviation 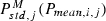 from the experiment shown in Fig. 2B and the 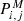 values predicted with kinetic parameters optimized according to Model I (A), Model II (B) and Model III (C). Shaded areas indicate systematic trend in the residual plot. Symbols show residuals for reactions with different initial substrate concentrations (in μm): 40 (circles), 60 (squares), 80 (dots), 100 (crosses), 200 (asterisks), 300 (diamonds), 600 (triangles).

**Fig. 5:**
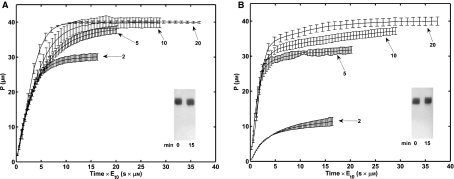
Selwyn's test of plasmin activity. The hydrolysis of Spectrozyme-PL (40 μm) by plasmin (at final concentrations, in nm, indicated by the numbers next to the curves) was monitored in reaction mixtures containing no other additive (A) or 115 μm stearate (B). The *p*-nitroaniline (P) generated is shown as the mean (lines) and standard deviation (cross-bars) of four measurements. Insets show silver-stained samples of plasmin (0.3 μm) incubated without (A) or with (B) 115 μm stearate at 37 °C for the indicated time in the presence of 70 μm Spectrozyme-PL and subjected to electrophoresis on a 10–15% polyacrylamide gel under nonreducing denaturing conditions.

Because, in most cases, the change in product concentration is not linear in the amidolytic assay performed with the required concentrations of plasmin and substrate, the initial reaction rate cannot be approximated reliably with a linear function, and the differential rate equation of the classic Michaelis–Menten framework cannot be applied directly for evaluating the experimental data. Accordingly, the progress curves of product generation in the course of the continuously monitored reactions were analysed as described in the Materials and methods. For the reactions in the absence of fatty acids, or in the presence of oleate and arachidonate, the experimental data were compatible with the simplest scheme of Model I (reversible substrate–enzyme interaction followed by irreversible breakdown of the enzyme–substrate complex to product and enzyme, as illustrated in Fig. 2A for oleate). In the presence of stearate, however, the discrepancy between the experimental and model curves was unacceptably large (Fig. 2B), suggesting a decrease in the enzyme activity during the assay. Consequently, the potential effects of the equilibrium between the product and the enzyme–product complex (Model II), as well as enzyme instability (Model III), are also considered, resulting in a decrease of the global-fit *χ*^2^ values (Fig. 3). Inspection of the residual plots [9] that were generated with the best estimates according to the three models evaluated, found systematic anomalies in Models I and II, which disappeared in Model III (Fig. 4). Selwyn's test [10] is a simple functional probe for enzyme stability in the course of activity assays. As illustrated in Fig. 5, this test indicated minimal loss of plasmin activity in the absence of fatty acids (Fig. 5A) compared with the markedly lower levels of end-stage product in the presence of stearate (Fig. 5B). In line with earlier observations [6], no autocleavage of plasmin was seen during the activity assay in the presence of enzyme substrate (Fig. 5, insets) precluding a proteolytic mechanism of the inhibition. The improved global *χ*^2^ value (Fig. 3), the homogeneous residual plot (Fig. 4) and the results of Selwyn's test (Fig. 5), justify the application of Model III as being the most adequate for the final evaluation of the kinetic parameters in the presence of stearate.

### Evaluation of kinetic parameters

**Fig. 6:**
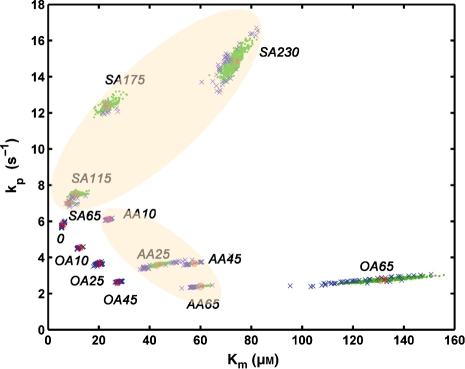
Kinetic parameters of plasmin in the presence of fatty acids. The values of *k*_p_ and *K*_m_ were determined from the amidolytic assay of plasmin activity, according to Model I, in the presence of oleate (*OA*) and arachidonate (*AA*), or, according to Model III, in the presence of stearate (*SA*). Using the Monte Carlo procedure described in the Materials and methods, 1000 synthetic sample sets were generated for each experimental setting and the estimated synthetic parameters are shown by green symbols (for pairs within the 95% confidence region; the exact numerical values are presented in Table 1) or blue symbols (for pairs out of the 95% confidence region). The best estimate from the experiment is indicated by a red asterisk, whereas the best estimate from the Monte-Carlo simulation is indicated by a red circle. Numbers following the abbreviation of the respective fatty acid name indicate its concentration (in μm). The ‘*0* ’ indicates the set of parameters in the absence of fatty acids. Shaded ellipses combine parameters belonging to the same type of fatty acid (data for oleate and the absence of fatty acids are found in the unshaded area).

**Fig. 7:**
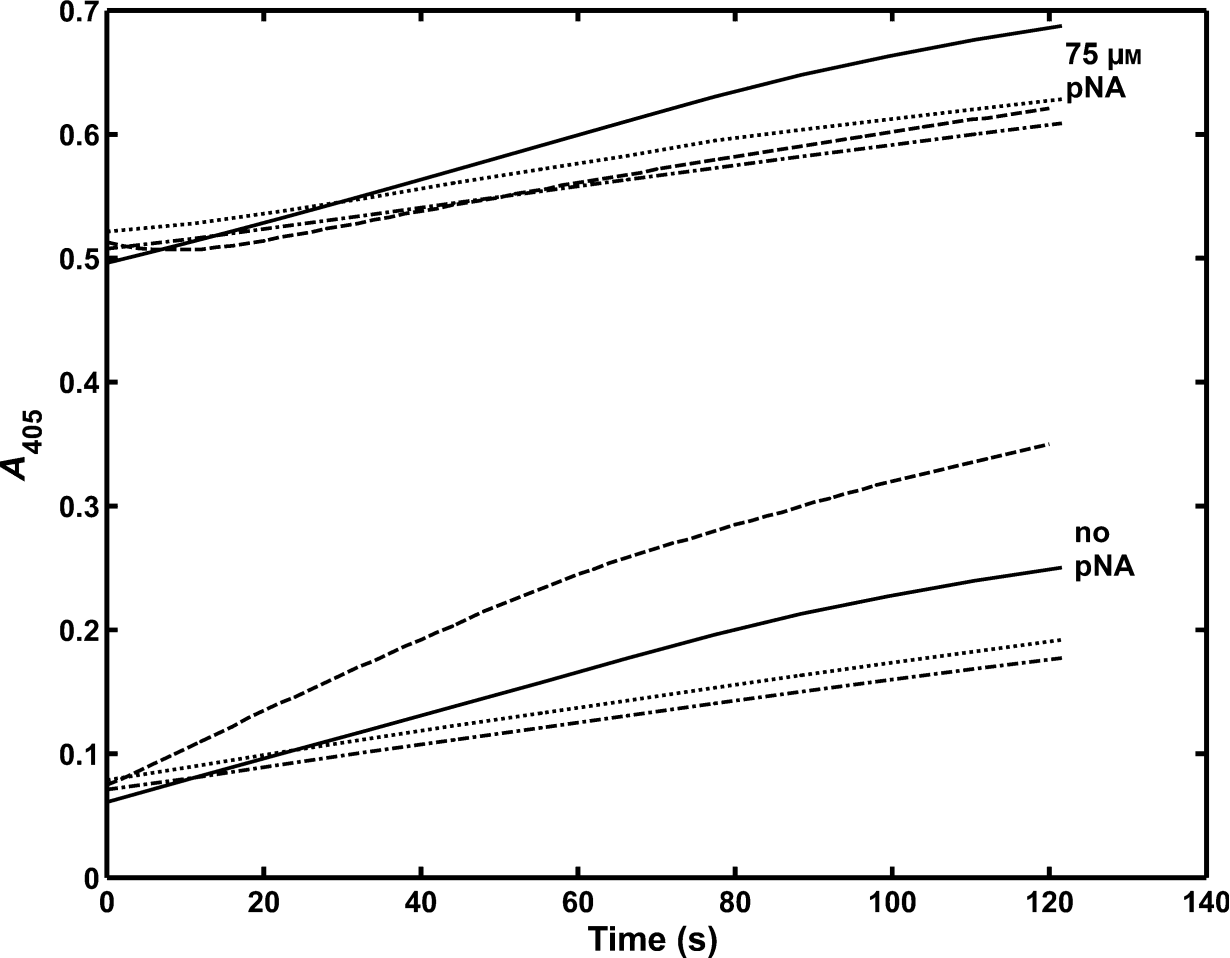
Effect of *p*-nitroaniline (pNA) on the amidolytic activity of plasmin in the presence of fatty acids. The absorbance at 405 nm was measured for reaction mixtures containing 20 nm plasmin, 80 μm Spectrozyme-PL and 115 μm stearate (dashed line), 45 μm oleate (dotted line), 45 μm arachidonate (dashed-and-dotted line) or no additive (solid line), in the absence or presence of pNA. The mean values of four measurements are presented.

Following preliminary estimates of the Michaelis constant *K*_m_, a specific range of Spectrozyme-PL concentrations was assigned for each concentration of each fatty acid (lower limit below the estimated *K*_m_ value and upper limit at least 5-fold higher than the *K*_m_ estimate). The final best estimates of the catalytic constant (*k*_p_) and the *K*_m_, and their confidence intervals, are presented in Fig. 6. The optimization according to Model III yielded three additional fitted parameters [the product–enzyme association equilibrium constant (*K*_i_), the decay rate constants for the enzyme-substrate complex (*J*_2_) and the enzyme product complex (*J*_3_)], which accounted for the progressive decrease of plasmin activity in the course of the assay in the presence of stearate (Table 1). The good fit of Model III progress curves to the experimental data supports the concept that in the presence of stearate the catalytic mechanism of plasmin is changed. The enzyme acquires higher affinity for some of the reaction products and, in addition, is less stable in complex with the product (the values of *J*_2_ assigned by the optimization procedure approach zero, thus ruling out potential instability of the enzyme–substrate complex). Additional experiments identified *p*-nitroaniline as the factor responsible for the premature decline of the enzyme activity in the course of the assay. The amidolytic assay was performed with plasmin pre-incubated with stearate and reaction products. If the pre-incubation was carried out with lysine, ε-aminocaproic acid or fibrinogen degradation products (mimicking the release of C-terminal lysine from Spectrozyme-PL) the time course of the amidolytic reaction was not affected, but if *p*-nitroaniline was used in the pre-incubation, the reaction started at the suppressed rate observed in the later stages of the amidolytic assay with stearate (Fig. 7). Thus, we concluded that the product inhibition was related only to the experimental setting of the amidolytic assay. Under such conditions the mathematical procedure operating with *K*_i_ and *J*_3_ is an indispensable tool in identification of the *k*_p_ and *K*_m_ values because it is able to eliminate the superimposed assay-dependent effects (on the *K*_m_ and *k*_p_), which is not a trivial problem.

**Table 1:** Kinetic parameters of plasmin in the presence of fatty acids. Numerical values of the best estimates (BE) and their 95% confidence intervals (CI) are presented. Values for the Michaelis constant (*K*_m_), (*k*_p_), the product–enzyme association equilibrium constant (*K*_i_) and the decay rate constant (*J*_3_) of the enzyme–product complex are presented, according to Model I, for no additive, oleate and arachidonate (NA indicates that the respective constant is not applicable in the used model) or according to Model III for stearate (decay rate constants of the enzyme–substrate complex are not shown because in all cases the optimization assigns them values of less than 10^−12^·s^−1^).

	*K*_m_ (μm)		*k*_p_ (s^−1^)		*K*_i_ (μm^−1^)		*J*_3_ (s^−1^)	
				
Concentration of added fatty acid (μm)	BE	CI	BE	CI	BE	CI	BE	CI
None	5.89	5.43–6.38	5.81	5.70–5.93	NA	NA	NA	NA
Oleate								
10	12.58	11.68–13.41	4.54	4.48–4.60	NA	NA	NA	NA
25	20.09	18.76–21.26	3.65	3.56–3.73	NA	NA	NA	NA
45	27.49	26.43–28.57	2.63	2.58–2.68	NA	NA	NA	NA
65	131.09	115.33–146.13	2.75	2.57–2.95	NA	NA	NA	NA
Arachidonate								
10	23.71	22.91–24.58	6.10	6.06–6.15	NA	NA	NA	NA
25	42.65	38.28–47.66	3.57	3.43–3.71	NA	NA	NA	NA
45	57.51	55.24–59.60	3.68	3.62–3.73	NA	NA	NA	NA
65	59.85	56.76–62.73	2.40	2.35–2.44	NA	NA	NA	NA
Stearate								
65	8.17	7.35–9.10	7.03	6.93–7.12	0.025	0.011–0.053	0.026	0.018–0.038
115	11.33	9.42–13.41	7.48	7.37–7.61	0.012	0.008–0.402	0.056	0.001–0.158
175	23.37	20.98–26.88	12.39	11.92–12.73	0.007	0.005–0.009	0.062	0.052–0.069
230	72.96	69.86–75.86	14.77	13.94–23.85	0.002	0.001–0.003	0.074	0.001–0.356

All three fatty acids examined caused a 10–20-fold increase in the *K*_m_ of plasmin: oleate and arachidonate were efficient in the 10–65 μm concentration range, whereas stearate needed to be present at concentrations higher than 65 μm to achieve significant effects (Fig. 6). The two unsaturated fatty acids (oleate and arachidonate) resulted in a decrease, of up to two-fold, in the *k*_p_ of plasmin. Considering the recently described reversible nature of the plasmin inhibition by oleic acid [3] and the changes of the kinetic parameters reported in the present study, oleate and arachidonate can be defined as mixed-type inhibitors of plasmin. The effect of stearate is rather unusual; the increase in the *K*_m_ is coupled to higher values of the *k*_p_. At saturating concentrations of the substrate this effect is seen as apparent activation of plasmin in the amidolytic assay. However, if we use the *k*_p_*/K*_m_ ratio as a measure of the overall impact of stearate, it should be classified as an inhibitor of plasmin, the potency of which is the lowest among the three fatty acids studied (Table 2).

**Table 2:** Catalytic efficiency [the catalytic constant/ Michaelis constant (*k*_p_*/K*_m_) ratio] of plasmin in the presence of various fatty acids. The *k*_p_*/K*_m_ (μm^−1^·s^−1^) ratio was calculated from the best estimates of the kinetic parameters for the amidolytic activity of plasmin presented in Fig. 6.

Fatty acid concentration(μm)	Stearate	Oleate	Arachidonate
0	0.99	0.99	0.99
10	–	0.36	0.26
25	–	0.18	0.08
45	–	0.10	0.06
65	0.86	0.02	0.04
115	0.66	–	–
175	0.53	–	–
230	0.20	–	–

### Structure–function relationships

**Fig. 8:**
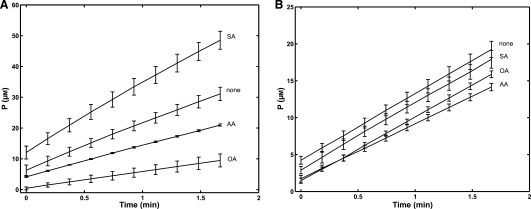
Amidolytic activity of des-kringle plasmin derivatives in the presence of fatty acids. The activity of 20 nm miniplasmin (A) and microplasmin (B) on 120 μm Spectrozyme-PL in the absence of additives (none) or in the presence of 45 μm oleate (OA), 45 μm arachidonate (AA) or 175 μm stearate (SA), was measured. The mean and standard deviation values of four measurements are presented.

In an attempt to identify the site of action of the fatty acids in the plasmin molecule, the amidolytic activity of two truncated plasmin variants was examined (Fig. 8). Miniplasmin (des-kringle 1-4 plasmin) contains the kringle 5 and the catalytic domain of plasmin, whereas microplasmin (des-kringle 1-5 plasmin) is composed of the catalytic domain only [11]. At a saturating concentration of Spectrozyme-PL, all three fatty acids affected the activity of miniplasmin in the same manner as that of plasmin; apparent activation was seen with stearate and inhibition was seen with oleate and arachidonate (Fig. 8A). Microplasmin was not sensitive to the presence of fatty acids (Fig. 8B). These results preclude the catalytic domain as a target of the fatty acids and support the notion that interaction with kringle 5 is sufficient for their action. This finding is in agreement with an earlier report that oleic acid binds to kringle 5 with an affinity that is an order of magnitude higher than found for binding to the other kringles [7]. Remarkably, the same authors show that oleic acid affects plasmin activity measured on a macromolecular substrate (prostromelysin-1) when applied in the same concentration range as reported in the present study for oleate and arachidonate (10–65 μm).

### Concluding remarks: advantages of progress curve analysis combined with Monte Carlo simulation

Our findings illustrate the general possibility for a modulator to change the kinetic parameters of an enzyme in an independent and controversial manner, so that the overall catalytic outcome may vary with the concentration of the substrate.

The approach used for the identification of kinetic parameters in this study exploited the advantages of progress curve evaluation in enzyme assays (a single reaction mixture yields 60 experimentally measured points for exactly the same enzyme and modulator concentrations) and further expanded the ideas for computer-intensive procedures in time-course simulations [12–14]. The global fit of the inverse functions for the integrated rate equations (Models I and II), or of the numerical solutions for the ordinary differential equations (Model III), was based on 420 experimental points (the means of 60 measured points for seven substrate concentrations) and its best estimates were further analyzed for better implementation of the experimental error. The application of error models 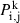 in the Monte Carlo simulations has the advantage over the real error in that it reflects the trend in the error as a function of the product concentration generated and filters the random effects in individual samples. The best estimates of the kinetic parameters differed slightly from the experimental estimates (Fig. 6) because they are actually corrected for the effect of random outliers. The probability distribution of the parameters estimated using this robust evaluation procedure was essential for the identification of the statistical significance of the described effects. In conclusion, our study is an example of a careful kinetic analysis that can be a valuable tool in the coherent interpretation of apparently controversial modulator effects on enzyme activity.

Our present results were gained in a homogeneous plasmin assay system and thus their pathophysiological implications are not straightforward with respect to external, therapeutic fibrinolysis [1], when plasmin is generated by plasminogen activators on preformed fibrin and is exposed to a constant substrate concentration in a narrow lysis front on the surface of fibrin. Even if intravascular events initiate blood clotting and fibrin dissolution simultaneously in a process called intrinsic or internal fibrinolysis [15], plasmin is generated directly on the surface of fibrin fibers and so it is partially protected against inhibitors [16]. Thus, the effects of fatty acids are restricted to a probably small fraction of plasmin molecules, which are detached from the fiber matrix. Despite this limitation, acting as mixed-type inhibitors, unsaturated fatty acids are still able to stabilize fibrin against plasmin, as previously reported for oleic acid [3], whereas through its discordant effects on the *k*_p_ and *K*_m_ values, stearate may promote fibrinogen depletion (as a result of higher plasmin activity at saturating substrate concentrations) and consequently shorten the life span of newly formed clots. The extrapolation of the reported *in vitro* effects to the *in vivo* setting of fibrin(ogen)olysis should await similarly rigorous characterization of plasmin activity on its natural substrates.

## Materials and methods

### Materials

Sodium salt and free acid forms of oleic, stearic and arachidonic acids were purchased from Sigma-Aldrich Kft (Budapest, Hungary) and stock solutions (10 mm) were prepared in water (prewarmed to 70 °C) containing 50 μm butylated hydroxytoluene; these stock solutions were further diluted to the desired concentrations in 10 mm HEPES buffer (pH 7.4) containing 150 mm NaCl (all reactions were performed in this buffer system, the butylated hydroxytoluene after the final dilution in the reaction mixtures had no effect on the plasmin activity on its own). Miniplasmin and microplasmin were prepared and titrated according to our previously published procedures [11].

### Amidolytic assay of plasmin activity

Plasmin (20 nm) was incubated with the sodium salts of fatty acids for 15 min at 37 °C. Then, 180 μL of this mixture was added to 20 μL of Spectrozyme-PL (H-D-norleucyl-hexahydrotyrosyl-lysine-*p*-nitroanilide; American Diagnostica, Stamford, CT, USA) at seven different concentrations ranging from 0.05 to 6 mm, yielding a final concentration *S*_0j_ (*j* = 1,2,...,7) in the volume of the reaction mixtures. The light absorbance at 405 nm (*A*_405_), which reflects the release of *p*-nitroaniline, was measured continuously at *t*_i_ (*i* = 1,2,...,60) time points in the course of 10 min at 37 °C; four parallel measurements were carried out for each *S*_0j_. The delay time between the initiation of the reaction and the first measurement was estimated with linear extrapolation from the initial six measured *A*_405_ values back to baseline absorbance, and an extinction coefficient for *p*-nitroaniline of 12.6 mm^−1^·cm^−1^ (determined from calibration in our assay system) was used to convert the measured absorbance values to product concentration 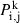 (the notation indicates the *p*-nitroaniline concentration at time *t*_i_ for the *k*th replica with *S*_0j_)*.* Using *P*_mean,i,j_ (mean of 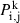) and *P*_std,i,j_ (standard deviation of 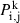) three different approaches to model the behaviour of the experimental error along the progress curves were tested (uniform error, linear regression in logarithmic scale and regression according to a truncated square root function). Among them, the best fit was achieved with the linearized logarithmic model equation 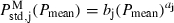, where 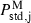 is the model error for the experiments with *S*_0j_. The parameters *a*_j_ and *b*_j_ were estimated by the ordinary least square method, and the experimental error models were used in evaluation of the kinetic parameters described below.

### Estimation of the kinetic parameters of plasmin according to different model mechanisms

Three different models were tested for the reaction catalyzed by plasmin in the aforementioned assay. In the simplest case (Model I) the scheme 
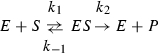
 is assumed, where *E* is plasmin, *S* is Spectrozyme-PL, *P* is *p*-nitroaniline, and *k*_1_, *k*_2_ and *k*_-1_ are the respective reaction rate constants. With the quasi-steady-state assumption the differential rate equation for this scheme is:
(1)
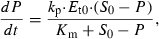

where *E*_t0_ and *S*_0_ are the initial concentrations of plasmin and its substrate, the Michaelis constant 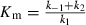 and the catalytic constant *k*_p_ = *k*_2_ [12]. Following integration it gives:
(2)
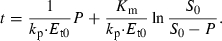


It is obvious that *t* in Eqn (2) is a strictly increasing function of *P* for any combination of *K*_m_ and *k*_p_ and therefore it has an inverse function *P* = *P*^M^(*t*, *K*_m_, *k*_p_, *S*_0_, *E*_t0_), which can be numerically estimated for all measured time points by a table look-up procedure. Thus, the linearized (according to the parameters) version of the integrated kinetic Eqn (2) was not used for regression purposes. With our approach we numerically built up the table of the inverse function for Eqn (2), which has no analytical form for *P* and in which *t* is the independent variable. Such multiple tables for different sets of parameters are used in the iterations, when the parameters are identified.

Because in the course of certain experiments the reaction rate declined faster than predicted by Model I, the more general scheme, 
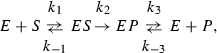
 was also tested (Model II), which accounts for the accumulation of the product and its complex with the enzyme. Assuming steady-state for both *ES* and *EP* complexes, the differential rate-equation is:
(3)
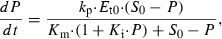

where 
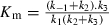
, 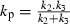 and the equilibrium association constant for the product 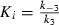. Although the *K*_m_ and the *k*_p_ derived for Model I and Model II have different algebraic form, their meaning within the context of the specific catalytic mechanism is identical; the *K*_m_ is the substrate concentration at which the initial reaction rate is half of the maximal rate possible for given enzyme concentration, whereas *k*_p_ has the properties of a first-order rate constant defining the capacity of the enzyme–substrate complex to form product [12]. The integrated form of Eqn (3) is:
(4)
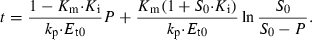


It can be proved that *t* in Eqn (4) is a strictly increasing function of *P* for any combination of *K*_m_, *k*_p_ and *K*_i_ and therefore it has an inverse function *P* = *P*^M^(*t*, *K*_m_, *k*_p_, *K*_i_, *S*_0_, *E*_t0_), which can be estimated numerically using the table look-up procedure described for Model I.

Because the product inhibition could not model the progress curve of the reaction in a satisfactory manner, the instability of the enzyme in the assay system was also considered according to the scheme suggested by Duggleby [13,14]

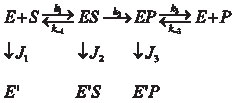

in which *E ′* indicates the inactive form of the enzyme, and *J*_1_, *J*_2_ and *J*_3_ are the rate constants for inactivation of the respective forms of the enzyme. In independent measurements with fatty acids we showed that the inactivation of free plasmin for the duration of the amidolytic assay was negligible (data not shown) and consequently the differential equation for the changes in enzyme concentration was derived only for *J*_2_ = 0, yielding the following system of ordinary differential equations (ODE):
(5)
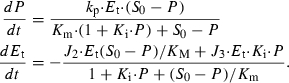


The ODE system (5) was solved with initial conditions *P*_(t=0)_ = 0 and *E*_(t=0)_ = *E*_t0_. The first component of the solution *P* = *P*^M^(*t*, *K*_m_, *k*_p_, *K*_i_, *J*_2_, *J*_3_, *S*_0_, *E*_t0_) represented the values for *P* in Model III. The integration of the ODE system (5) was performed by quasi-constant step-size implementation in terms of backward differences of the Klopfenstein–Shampine family of Numerical Differentiation Formulas of orders 1–5 and the initial steps were determined so that the solution would stay in its domain (0 ≤ *P* ≤ *S*_0_, 0 ≤ *E*_t_ ≤ *E*_t0_) during the whole integration [17].

The model equations were fitted to the
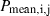
values with minimization of the square residues. The best experimental estimate of the model parameters was defined as the set of parameters for which the 
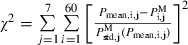
 was rendered the minimal value 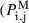 is the value at *t*_i_ and *S*_0j_ of the functions with different sets of kinetic parameters as defined for Models I, II or III above and an identical *E*_t0_ value for all experiments). The minimization was performed using the Nelder–Mead simplex direct search method [18]. Monte Carlo simulations [19] over the parameter logarithms were used to identify the confidence intervals of the parameters and their best estimates, as described previously [20]. Each value for the simulated data points in the synthetic sample set was generated as a random entry, chosen from the normal distribution with mean *P*_mean,i,j_ and variance 
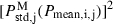
. All model simulation and optimization programs described above run under matlab 7.4 (The MathWorks Inc., Natick, MA, USA).

## Abbreviation

ODE: ordinary differential equation
